# High temperature increases centromere-mediated genome elimination frequency and enhances haploid induction in *Arabidopsis*

**DOI:** 10.1016/j.xplc.2022.100507

**Published:** 2022-12-20

**Authors:** Ulkar Ahmadli, Manikandan Kalidass, Lucie Crhak Khaitova, Joerg Fuchs, Maria Cuacos, Dmitri Demidov, Sheng Zuo, Jana Pecinkova, Martin Mascher, Mathieu Ingouff, Stefan Heckmann, Andreas Houben, Karel Riha, Inna Lermontova

**Affiliations:** 1 Leibniz Institute of Plant Genetics and Crop Plant Research (IPK) Gatersleben, Corrensstrasse 3, 06466 Seeland, Germany; 2 Central European Institute of Technology (CEITEC) and National Centre for Biomolecular Research, Faculty of Science, Masaryk University, Kamenice 5, 625 00 Brno, Czech Republic; 3 CIRAD, DIADE, IRD, University of Montpellier, 34393 Montpellier, France

**Keywords:** centromere, kinetochore null 2, CENPC-k, *cenh3-4*, temperature stress, haploid induction

## Abstract

Double haploid production is the most effective way to create true-breeding lines in a single generation. In *Arabidopsis*, haploid induction via mutation of the centromere-specific histone H3 (cenH3) has been shown when the mutant is outcrossed to the wild-type, and the wild-type genome remains in the haploid progeny. However, factors that affect haploid induction are still poorly understood. Here, we report that a mutant of the cenH3 assembly factor Kinetochore Null2 (KNL2) can be used as a haploid inducer when pollinated by the wild-type. We discovered that short-term temperature stress of the *knl2* mutant increased the efficiency of haploid induction 10-fold. We also demonstrated that a point mutation in the CENPC-k motif of KNL2 is sufficient to generate haploid-inducing lines, suggesting that haploid-inducing lines in crops can be identified in a naturally occurring or chemically induced mutant population, avoiding the generic modification (GM) approach at any stage. Furthermore, a *cenh3-4* mutant functioned as a haploid inducer in response to short-term heat stress, even though it did not induce haploids under standard conditions. Thus, we identified *KNL2* as a new target gene for the generation of haploid-inducer lines and showed that exposure of centromeric protein mutants to high temperature strongly increases their haploid induction efficiency.

## Teaser

The production of haploids (double haploids) is used to accelerate crop breeding. This study reports that a mutant of the centromere-specific histone H3 assembly factor KNL2 induces haploid formation when crossed with the wild-type. It also shows that short-term temperature stress of the *knl2* mutant increases its haploid induction efficiency up to 10-fold.

## Introduction

Haploid generation technology, followed by whole-genome duplication, is an effective strategy for accelerating plant breeding because it enables the production of true-breeding lines with complete homozygosity in a single step. In the conventional breeding approach, these lines are obtained by inbreeding, and seven to nine generations of inbreeding are often performed over several years to achieve the desired level of homozygosity (Britt and Kuppu, 2016). Two main approaches have been widely used to produce (double) haploids (DHs): the *in vitro* explantation of gametophytic tissues (mainly cultivation of anthers or microspores) and the selective loss of one parental chromosome set *in vivo* through interspecific or intraspecific hybridization (Kalinowska et al., 2019). However, depending on the tissue culture or crossing ability of the species of interest, these approaches can be applied to only a limited number of genotypes. Hence, alternative resource-efficient and reliable approaches for DH production are urgently needed. One way to improve DH effectiveness is to develop efficient inducer lines that guarantee a high haploid induction rate (HIR) combined with a high-throughput haploid selection system. One promising approach for haploid induction is the use of centromere-mediated genome elimination (Ravi and Chan, 2010; Kuppu et al., 2015).

Centromeres are unique chromosomal regions that mediate kinetochore protein complex formation and microtubule attachment during cell division (Verdaasdonk and Bloom, 2011; Schalch and Steiner, 2017). Most centromeres are epigenetically defined by nucleosomes containing the centromere-specific histone H3, cenH3 (Allshire, 1997). The cenH3 protein contains two domains, an N-terminal tail, which is a target for post-translational modification, and a C-terminal histone fold domain, which interacts with DNA and other histones to form the nucleosome. The loading of cenH3 to centromeres initiates assembly of the functional kinetochore complex. The cenH3 loading pathway can be divided into three steps: initiation (centromere licensing), deposition, and maintenance. The centromere licensing factor KNL2 (Kinetochore Null2), identified in *Arabidopsis thaliana*, showed colocalization with cenH3 throughout the cell cycle except from metaphase to mid-anaphase (Lermontova et al., 2013). Furthermore, Sandmann et al. (2017) identified a cenH3 nucleosome-binding CENPC-k motif at the C-terminal domain of KNL2. Either the complete deletion of this motif or mutation of its conserved amino acids abolished the localization of KNL2 at centromeres. Thus, it is evident that the CENPC-K motif is functionally required for centromeric localization of KNL2 in *A. thaliana* (Sandmann et al., 2017).

Because of its essential function in chromosome segregation, inactivation of cenH3 has been shown to result in chromosome segregation errors and lethality (Ravi and Chan, 2010; Ravi et al., 2011). RNAi-mediated knockdown of *cenH3* led to a reduction in its mRNA level (27%–43%) and also resulted in a dwarf plant phenotype and meiotic defects in *Arabidopsis* (Lermontova et al., 2011). Recently, a mutation in cenH3 named *cenh3-4* was discovered from a genetic suppressor screen and shown to increase fertility and promote meiotic exit in *smg7-6* plants (Capitao et al., 2021). The *cenh3-4* mutant contains a point mutation (G→A) in the third exon of cenH3. It showed a reduced amount of cenH3 at the centromeres and, thus, formed small centromeres. Similar to the cenH3 RNAi transformants, a transfer DNA (T-DNA) insertion knockout mutant of KNL2 showed a reduced amount of cenH3 at the centromeres, decreased growth rate, decreased fertility, and meiotic defects (Lermontova et al., 2013), further supporting the functional relationship of both proteins.

Ravi and Chan (2010) discovered that haploid plants can be obtained by pollination of a *cenh3-1* mutant of *A. thaliana* complemented with a GFP-tail swap construct (fusion of the N terminus of conventional H3 to the C terminus of cenH3) with different wild-type accessions. This cross resulted in haploid progenies that contained the genome of the wild-type parent at frequencies as high as 25%–45%. If a wild-type female was crossed to a GFP-tail swap male, the proportion of haploid plants was lower. In recent studies, haploids were also obtained by introducing point mutations or small deletions in *Arabidopsis* cenH3 (Karimi-Ashtiyani et al., 2015; Kuppu et al., 2015, 2020). Marimuthu et al. (2021) showed that cenH3 variants complementing the *cenh3-1* mutant are selectively removed from centromeres during reproduction. In addition, the authors demonstrated that the null mutant of VIM1 (VARIANT IN METHYLATION 1) enhances the haploid induction frequencies of the complemented *cenh3-1* mutant.

The cenH3-based haploid induction approach was successfully extended from *Arabidopsis* to crop plants, but use of the homozygous *cenh3* mutant complemented with an altered variant of cenH3 resulted in an average haploid induction frequency below 1% in maize (Kelliher et al., 2016). However, it was recently reported that use of a heterozygous cenH3 null mutation increased the HIR in maize to 5% (Wang et al., 2021). Application of a similar haploid induction approach to wheat resulted in an HIR up to 8% (Lv et al., 2020).

In *Arabidopsis*, haploid induction has been established by crossing a *cenH3* mutant with the wild-type. However, whether altering cenH3 assembly factors might also result in haploid induction has not yet been investigated. Furthermore, the conditions that may increase haploid induction remain elusive. In this study, we show that a T-DNA knockout mutant of KNL2 is an effective haploid inducer when crossed with *Arabidopsis* wild-type plants. We demonstrate that short-term exposure of *knl2* to heat stress leads to an increase in haploid induction efficiency from 1% to 10%. Moreover, the stress treatment regimen developed for the haploid induction process with the *knl2* mutant also appeared to be effective for the *cenh3-4* mutant. In addition, we showed that introduction of a point mutation in the CENPC-k motif of KNL2 is sufficient to create a haploid-inducer line.

## Results

### Short-term temperature stress of the *knl2* mutant increases the efficiency of haploid induction

**Figure 1:**
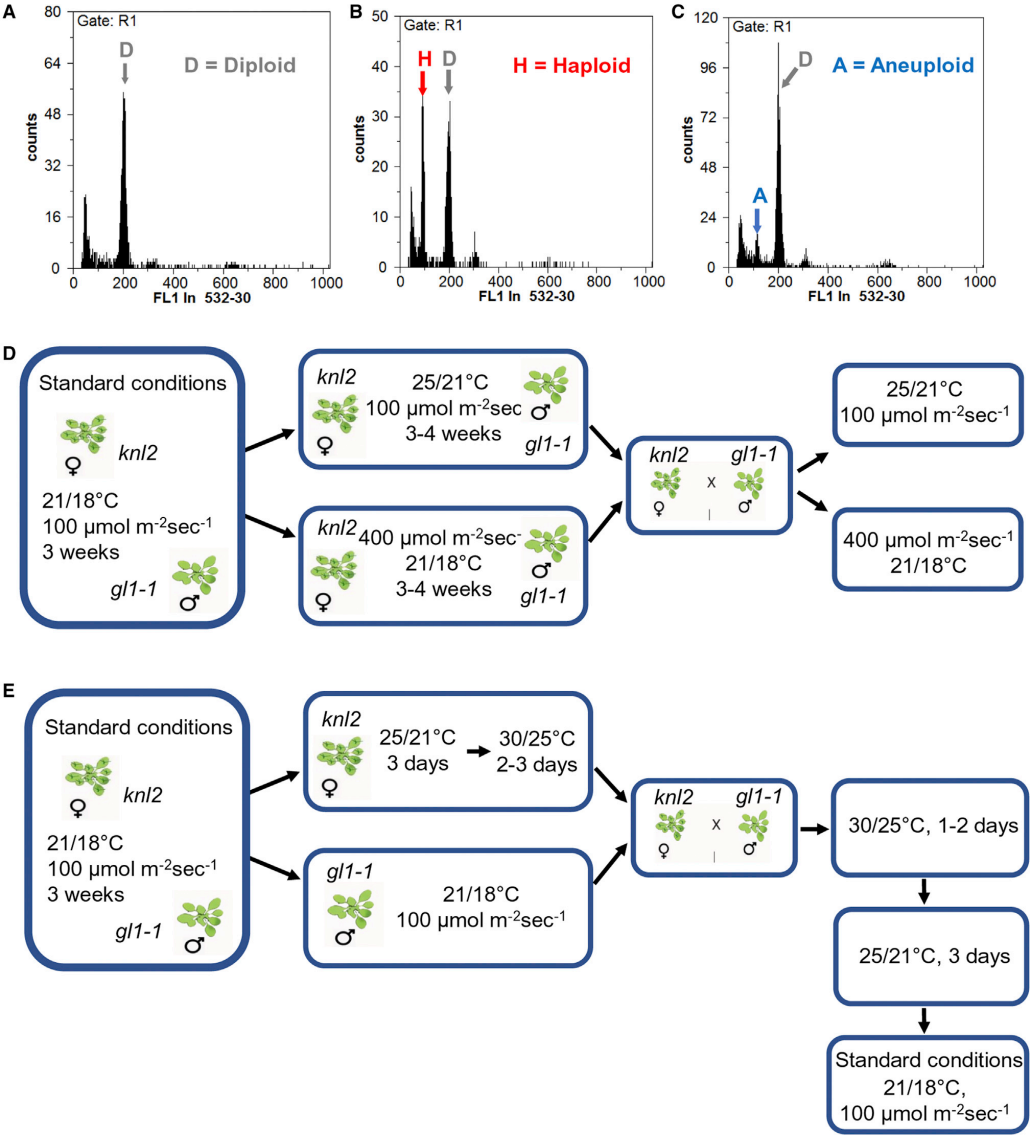
FC histograms of seed pools and the crossing schemes of the *knl2* mutant with the *gl1-1* marker line under stress conditions. **(A–C)**FC histogram of six-seed pools containing only diploid **(A)**, haploid and diploid **(B)**, and aneuploid and diploid **(C)** seeds. The presence of haploid/aneuploid seeds in the pools was assessed using the linear scale of the PI fluorescence intensity. In cases in which haploids or aneuploids were detected, only one of the six seeds was considered as deviating from the diploid status independent of their nuclei number. In comparison with the diploid population, just one haploid per pool has been evaluated from the six seed pools. As a result, the number of haploids/aneuploids may be underestimated. **(D)** The *knl2* and *gl1-1* plants were grown under standard growth conditions (21°C/18°C day/night and 100 μmol m^−2^ s^−1^ light intensity) for 3 weeks; then the *knl2* mutant plants and *gl1-1* plants were transferred to a growth chamber with constant higher temperature (25°C/21°C day/night) or light intensity (400 μmol m^−2^ s^−1^) for 3–4 weeks. Independent crossing of *knl2* and *gl1-1* was performed for increased temperature and light intensity, and the plants remained at the same conditions. **(E)** Similarly, *knl2* and *gl1-1* plants were grown under standard growth conditions until flowering. *knl2* plants were moved to high temperatures (25°C/21°C day/night) for 3 days followed by short-term temperature stress (30°C/25°C day/night) for 2–3 days, whereas the *gl1-1 L. erecta* marker line remained under standard growth conditions. Then the *knl2* and *gl1-1* plants were crossed and placed back in 30°C/25°C day/night for 1–2 days. The temperature was reduced stepwise: first to 25°C/21°C day/night for 3 days and then to standard conditions.

T-DNA knockout mutation of the cenH3 loading factor KNL2 (*knl2* mutant) results in a decreased amount of cenH3 protein, suggesting an essential role for KNL2 in the loading of cenH3 at the centromeres (Lermontova et al., 2013). Therefore, we assumed that crossing of the *knl2* mutant with wild-type *Arabidopsis* might generate haploids similarly to the cenH3-based haploid induction process. To test this hypothesis, we crossed the *knl2* mutant reciprocally with the *Arabidopsis* wild-type accession *Landsberg erecta* grown under standard conditions (STs). Flow cytometric (FC) analysis of pools of up to six seeds revealed 1% haploid progeny when *knl2* was used as the female parent (Figure 1A–1C and Table 1).

**Table 1:** Analysis of haploid induction by *knl2* and *cenh3-4* mutants based on ploidy levels measured by FC.

Cross (♀ × ♂)	Total seeds	No. of haploids	Haploids (%)	No. of aneuploids	Aneuploids (%)	Conditions
*knl2 ×* Ler	196	2	1	_1_	0.5	standard conditions
Ler *× knl2*	335	0	0	0	0	standard conditions
*knl2 × gl1-1*	200	0	0	1	0.5	25°C, 100 μmol m^−2^ s^−1^^a^
*knl2 × gl1-1*	108	1	0.9	1	0.9	21°C, 400 μmol m^−2^ s^−1^^a^
*gl1-1 × knl2*	126	0	0	0	0	21°C, 400 μmol m^−2^ s^−1^^a^
*knl2 × gl1-1*	256	19	7.4	8	5	30°C^b^
*gl1-1 × knl2*	108	0	0	0	0	30°C^b^
Col-0 *× gl1-1*	96	0	0	0	0	30°C^b^
*KNL2gen(W-R) Line 1 × gl1-1*	126	1	0.8	2	1.5	30°C^b^
*KNL2gen(W-R) Line 2 × gl1-1*	108	6	5.6	4	3.7	30°C^b^
*KNL2gen(W-R) Line 4 × gl1-1*	120	1	0.8	2	1.7	30°C^b^
*KNL2gen Line1 × gl1-1*	60	0	0	0	0	30°C^b^
*KNL2gen Line2 × gl1-1*	108	0	0	0	0	30°C^b^
*KNL2gen Line3 × gl1-1*	54	0	0	0	0	30°C^b^
*cenh3-4 × gl1-1*	336	11	3.3	9	2.7	30°C^b^
*gl1-1 × cenh3-4*	90	0	0	1	1.1	30°C^b^
*cenH3 RNAi × gl1-1*	120	0	0	0	0	30°C^b^
*gl1-1 × cenH3 RNAi*	66	0	0	0	0	30°C^b^

Plants carrying the mutant allele treated under different conditions were pollinated by either a wild-type Ler or *gl1-1* pollen donor. The *knl2* and *cenh3-4* mutants were published previously (Lermontova et al., 2013; Capitao et al., 2021). Haploid induction crosses between *knl2* and wild-type Ler or *knl2* and *gl1-1*, as well as crosses between *cenh3-4* and *gl1-1*, were performed at least three times. Seeds from the different crosses were combined together. One portion was analyzed by FC (Table 1); the other portion was germinated, and the haploids were scored based on the glabrous phenotype (Table 2). Haploid induction crosses between the *knl2* mutant complemented with wild-type KNL2 or KNL2 carrying the W-R mutation in the CENPC-k motif with *gl1-1* and crosses between the cenH3 RNAi line with *gl1-1* were performed once, and seeds were analyzed only by FC.

a Plants subjected to prolonged temperature or light stress (Figure 1D).

b Plants subjected to short-term temperature stress (30°C) (Figure 1E).

Our previous RNA sequencing (RNA-seq) data analysis revealed that a large number of stress-responsive genes are differentially expressed in *knl2* seedlings and flower buds compared with those of the wild-type (Boudichevskaia et al., 2019). We therefore hypothesized that *knl2* mutant plants may be more sensitive to stress treatment than control plants and that exposure of *knl2* to stress may increase HIR in its crosses with the wild-type. To support this assumption, we analyzed the expression of genes encoding cenH3 and the cenH3 assembly factors KNL2, CENP-C, and NASP under different stress conditions. The gene expression data were retrieved from the *Arabidopsis* transcriptome data platform (http://ipf.sustech.edu.cn/pub/athrdb/). The results showed that these genes were significantly downregulated in response to various stress treatments such as heat, 1-naphthylphthalamic acid, and fluctuating light (Supplemental Table 1 and Supplemental Figure 1). Thus, increased temperature and light intensity have a strong effect on the expression of genes encoding cenH3, KNL2, and other key kinetochore components.

To test the effect of stress on *knl2* growth and development and the induction of haploids, we exposed the mutant to high temperature or high light intensity before crossing. Use of the *gl1-1* mutant as a crossing partner enables the identification of haploids or DHs on the basis of the trichome-less phenotype (Kuppu et al., 2015). First, all plants were cultivated for 3 weeks under long-day STs at day/night temperatures of 21°C/18°C and a light intensity of 100 μmol m^−2^ s^−1^. One set of plants then remained under standard growth conditions, whereas others were transferred to either higher temperatures (25°C/21°C day/night) or high light intensity (400 μmol m^−2^ s^−1^) (Figure 1D). For each growth condition, about 25 *knl2*, 15–20 wild-type, and 15–20 *gl1-1* plants were cultivated. At higher temperatures or light intensity, the phenotypic difference between the wild-type and the *knl2* mutant became more pronounced than under standard growth conditions (Supplemental Figure 2A–2C). Reciprocal crosses were performed between *knl2* mutant plants and *gl1-1* cultivated under the growth conditions described above. FC analysis of seed pools or trichome phenotype analysis of F1 plants revealed no increase in haploid induction efficiency under either type of continuous stress condition (Tables 1 and 2).

**Table 2:** Phenotype-based selection of haploid plants derived from crosses of *knl2* and *cenh3-4* with *gl1-1*.

Cross (♀ × ♂)	Total no. of plants	No. of haploids	Haploids (%)	Conditions
*knl2 × gl1-1*	144	0	0	standard conditions
*gl1-1 × knl2*	120	0	0	standard conditions
*knl2 × gl1-1*	144	0	0	25°C, 100 μmol m^−2^ s^−1^^a^
*gl1-1 × knl2*	92	0	0	25°C, 100 μmol m^−2^ s^−1^^a^
*knl2 × gl1-1*	144	1	0.7	21°C, 400 μmol m^−2^ s^−1^^a^
*gl1-1 × knl2*	68	0	0	21°C, 400 μmol m^−2^ s^−1^^a^
*knl2 × gl1-1*	114	12	10.5	30°C^b^
*gl1-1 × knl2*	29	0	0	30°C^b^
*cenh3-4 × gl1-1*	96	4	4.1	30°C^a^

Mutant plants cultivated under standard conditions and subjected to different stress conditions were crossed with the *gl1-1* pollen donor. The haploids were scored using the glabrous phenotype.

a Plants subjected to prolonged temperature or light stress (Figure 1D).

b Plants subjected to short-term temperature stress (30°C) (Figure 1E).

Assuming that we did not obtain an increase in HIR because of adaptation of the *knl2* mutant to continuous stress, the experimental setting was changed, and *knl2* mutant plants were exposed to high temperature (30°C) for only a short period (2–3 days) before crossing (Figure 1E) to impair gamete development and another 1–2 days after crossing to impair embryo development. The temperature was increased and decreased stepwise, as shown in Figure 1E, whereas the *gl1-1* crossing partner remained under STs. The reciprocal crosses were repeated at least three times in two different growth chambers. FC analysis of seed pools and *gl1-1* mutant phenotype analysis of F1 plants revealed similar haploid induction efficiencies of 7.4% and 10%, respectively, when heat-stressed *knl2* was used as the female parent. No haploids were detected when heat-stressed *knl2* was used as the pollen donor (Tables 1 and 2).

**Figure 2:**
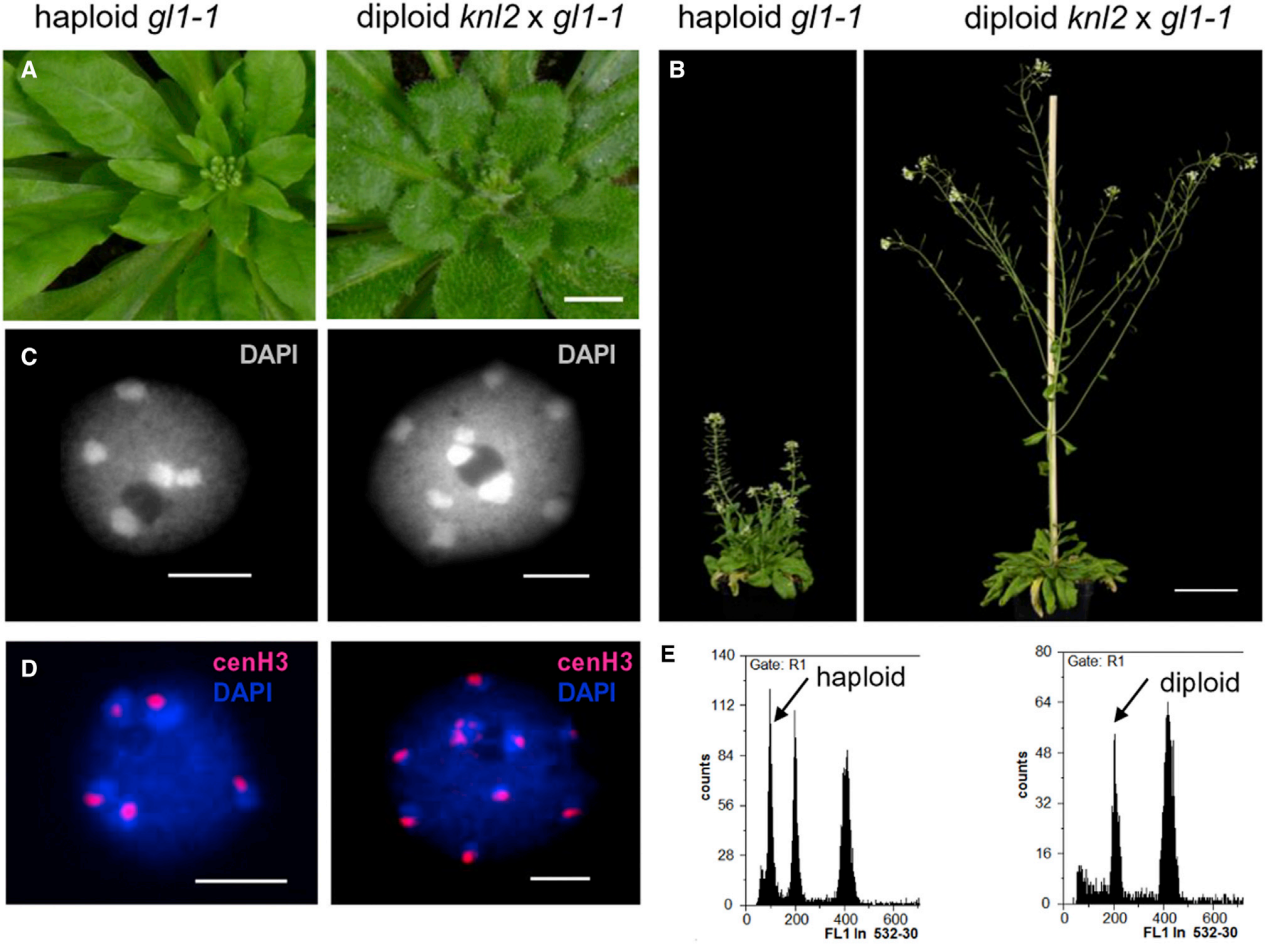
Haploid progenies obtained by genome elimination in crosses of *knl2* with the trichome-less *gl1-1* marker line. **(A)** Comparison of a haploid plant without trichomes (left) and the diploid hybrid phenotype with trichomes (right). Scale bar, 1 cm. **(B)** Phenotype of haploid *gl1-1* and diploid *knl2* × *gl1-1* hybrid plants during the generative development stage. Scale bar, 5 cm. **(C)** DAPI-stained nuclei isolated from haploid and diploid plants showing a maximum of 5 and 10 heterochromatic chromocenters, respectively. Scale bars, 5 μm. **(D)** Anti-cenH3-labeled nuclei isolated from haploid and diploid plants showing a maximum of 5 and 10 immunosignals (in red), respectively. Scale bars, 5 μm. **(E)** Histogram analysis of nuclei by FC for a *gl1-1* haploid offspring and a diploid control.

The trichomeless plants were much smaller than the corresponding diploids (Figure 2A and 2B). The 1C nuclei of selected *gl1-1* plants contained a maximum of five chromocenters (Figure 2C), and immunostaining of cenH3 also showed five chromocenter-localized signals, thus confirming haploidy (Figure 2D). A sample flow histogram plot of haploids produced from *knl2* and *gl1-1* crosses and a diploid control is shown in Figure 2E. In addition to haploids, 5% aneuploid seeds were detected by FC (Table 1 and Figure 1C). Control crosses of wild-type Col-0 with *gl1-1 Ler* under the same conditions did not produce haploid plants. Thus, short temperature stress increased haploidization frequency if *knl2* was used as a female crossing partner.

### A point mutation at the CENPC-k motif of KNL2 results in haploid induction on outcrossing

**Figure 3:**
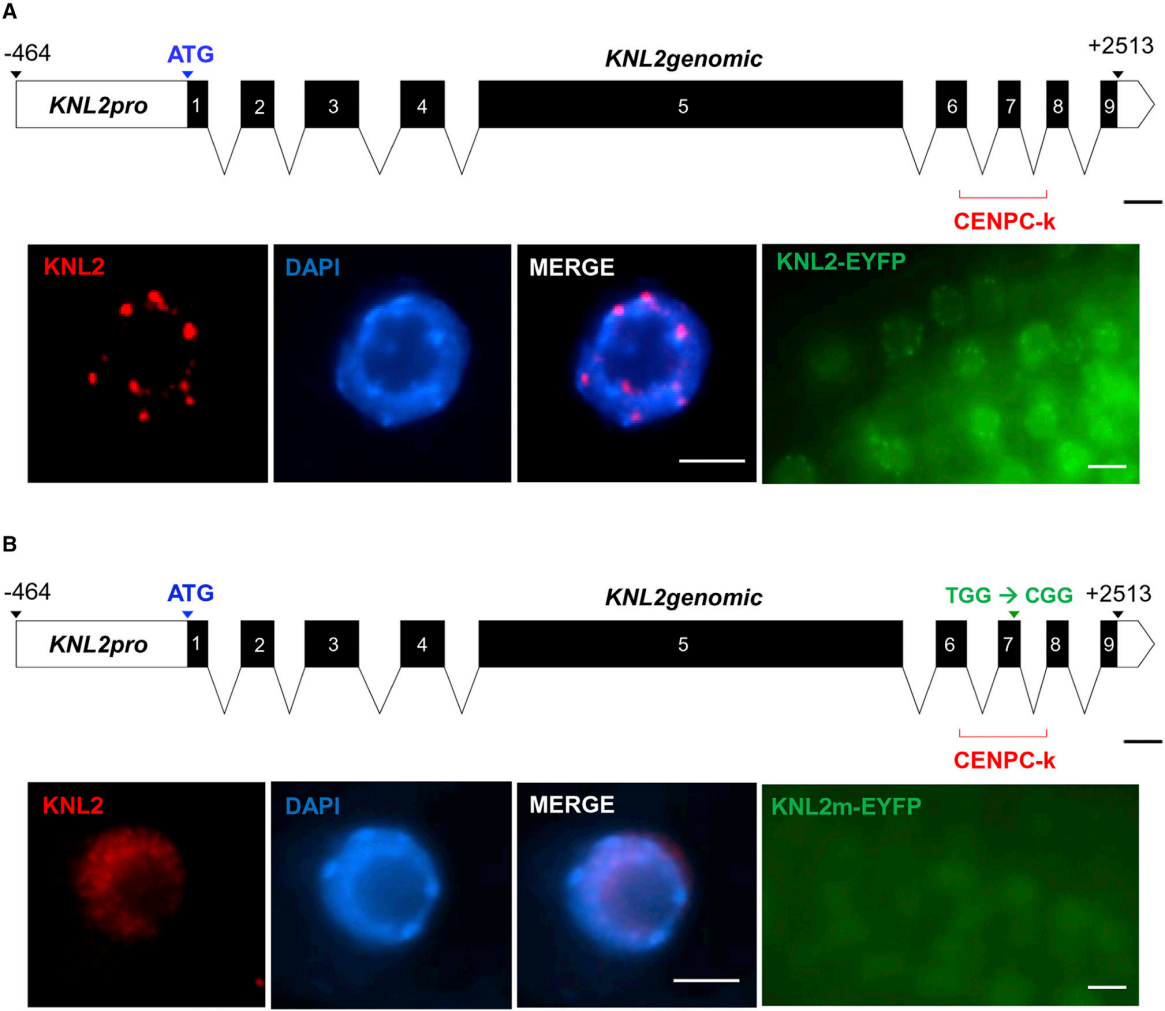
Substitution of the amino acid Trp by Arg within the conserved CENPC-k motif abolished the centromeric localization of KNL2. Schematic representation of the genomic KNL2 fragment with native promoter (upper panels) unmodified **(A)** or carrying the W-to-R mutation (TGG to CGG) within the CENPC-k motif **(B)** and subcellular localization of the corresponding fusion proteins in root tip nuclei of *Arabidopsis* immunostained with anti-KNL2 antibodies (first three lower left panels) or analyzed by confocal microscopy (lower right panel). The unmutated KNL2-EYFP fusion protein showed centromeric and nucleoplasmic localization **(A)**, whereas the protein with the point mutation was detected only in the nucleoplasm **(B)**. Scale bars represent 100 bp (upper panels) or 5 μm (lower panels).

We previously identified a conserved CENPC-k motif in the KNL2 protein and showed that deletion of this motif or mutagenesis of its conserved amino acids Arg-546 and Trp-555 abolishes its centromeric localization (Sandmann et al., 2017). To test whether the introduction of a point mutation into the CENPC-k motif would be sufficient for haploid induction, we cloned the genomic *KNL2* fragment with the endogenous promoter into the pDONR221 vector. To substitute the conserved Trp-555 with Arg, we performed PCR-based site-directed mutagenesis (Figure 3). The resulting mutant *knl2* and the wild-type *KNL2* were subcloned into the pGWB640 vector in fusion with EYFP and used for transformation of the *knl2* mutant. The selected transgenic plants were analyzed to determine the subcellular localization of the KNL2-EYFP fusion protein. In the *knl2* mutant complemented with the unmodified KNL2-EYFP construct, fluorescence signals were detected in the nucleoplasm and at chromocenters (Figure 3A), whereas in *knl2* expressing KNL2-EYFP with the point mutation in the CENPC-k motif, EYFP signals were detected only in the nucleoplasm (Figure 3B). Immunostaining of root tip nuclei from both transformant types with anti-KNL2 confirmed the centromeric localization of wild-type KNL2 and the nucleoplasmic localization of the mutant protein (Figure 3A and 3B). Three transgenic lines per construct were selected as female crossing partners for a haploid induction experiment with 30°C short-term temperature stress. The haploid induction efficiency of the *knl2* mutant complemented by KNL2-EYFP with the point mutation varied from 0.8% to 5.6%. By contrast, no haploids were detected in the case of *knl2* expressing the wild-type KNL2 control construct (Table 1).

### Analysis of paternal haploid plants did not reveal any traces of the maternal genome

Next, a PCR-based marker analysis of three DH plants was performed to confirm whether only the chromosomes of the pollen donor remained. One genotype-specific marker per chromosome was used (Supplemental Figure 3), and in all cases, PCR amplicons were found to correspond to the *gl1-1* mutant. To exclude the presence of small chromosome fragments as a byproduct of the haploidization process as reported by Tan et al. (2015), we performed a single-nucleotide polymorphism (SNP) analysis based on next-generation sequencing reads of DNA samples isolated from three DHs, one hybrid, and two parental plants. The SNP analysis clearly showed that the *gl1-1* DH plants did not contain parts of the maternal *knl2* chromosome complement (Supplemental Figure 4). The hybrid, by contrast, was heterozygous throughout its genome. A read-depth analysis did not show any chromosomal aberrations, as observed by Tan et al. (2015) (Supplemental Figure 5).

### Plants exposed to high temperature show reduced seed setting as a result of increased mitotic and meiotic abnormalities

In *Arabidopsis* wild-type and the *knl2* mutant, exposure to the high-temperature (30°C) regimen above (Figure 1E) resulted in reduced seed setting and an increased number of aborted seeds after selfing. However, this effect was more pronounced in the *knl2* mutant than in the wild-type (Supplemental Figure 6): after exposure to high temperature, the average seed number per silique was reduced from 53 to 33 in Col-0 and from 45 to 14 in the *knl2* mutant, and the number of aborted seeds was increased from 2 to 4 in Col-0 and from 5 to 9 in the *knl2* mutant. To determine whether the reduction in fertility was based on meiotic defects, we performed male meiotic chromosome spread analysis in wild-type and *knl2* plants exposed to the same growth conditions described above.

**Figure 4:**
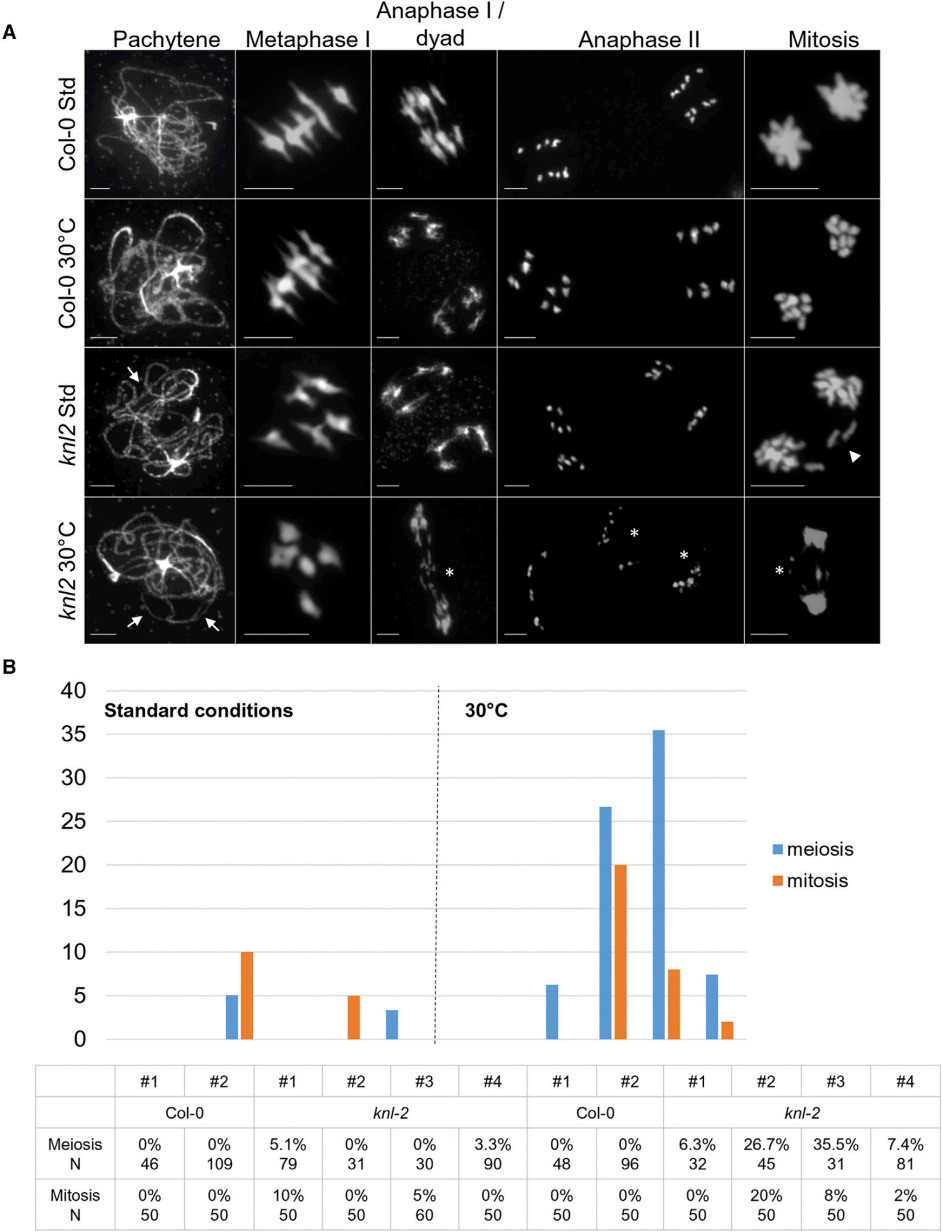
Mitotic and meiotic defects under high temperature in the *knl2* mutant. **(A)** Male meiosis and mitosis (from tapetum cells) in wild-type and *knl2* plants grown at 21°C or exposed to 30°C, as indicated in Figure 1E. Wild-type plants grown at either temperature showed no errors in the progress of meiosis and mitosis. During meiosis, homologous chromosomes undergo synapsis at pachytene, form five bivalents at metaphase I, segregate to opposite poles at anaphase I/dyad, and separate into sister chromatids during anaphase II. During mitosis, sister chromatids separate to opposite poles. In *knl2* plants, meiotic and mitotic defects are found, including asynapsis and interlocks during pachytene (arrows), lagging chromosomes (arrowhead), and chromosome fragmentation (asterisks) during mitotic and meiotic divisions. The chromosomes were stained with DAPI (blue). Scale bars, 5 μm. **(B)** The percentage of observed cells with defects per independent plant (meiosis: synapsis defects, lagging chromosomes, and chromosome fragmentation; mitosis: lagging chromosomes, anaphase bridges, and chromosome fragmentation) and the numbers of cells analyzed are indicated.

No meiotic defects were observed at 21°C or 30°C (two plants each) in wild-type plants. Homologous chromosomes undergo synapsis at pachytene, five bivalents are inevitably found at metaphase I, homologous chromosomes segregate during the first meiotic division, and the sister chromatids are separated during the second meiotic division (Figure 4A). Meiotic defects were detected in *knl2* plants grown at 21°C (two of four plants) and 30°C (all four plants), including synapsis defects (asynapsis and interlocks), as well as lagging chromosomes and chromosome fragmentation during the first and second meiotic divisions (Figure 4A). The degree of observed defects varied among plants and was more pronounced at 30°C than at 21°C (Figure 4B). Because of the observed meiotic chromosome fragmentation, mitotic divisions of tapetum cells from the same plants were also studied. Consistent with the meiotic defects, mitotic defects were also observed in *knl2* plants grown at 21°C (two of four plants) and 30°C (three of four plants), including lagging chromosomes, anaphase bridges, and chromosome fragmentation, and they were more pronounced at a higher temperature (Figure 4A and 4B). No obvious mitotic defects were found in the wild-type at either temperature (two plants each). In summary, varying degrees of mitotic and meiotic defects were found in *knl2* plants and were more frequent in plants grown at 30°C, whereas no noticeable defects were observed in wild-type plants under either temperature regimen.

### A *cenh3-4* mutation induces haploid formation under temperature stress

The *cenh3-4* mutant, which contains a point mutation in cenH3 (G→A substitution in the splicing donor site of the third exon of cenH3), showed a substantially reduced level of cenH3 at the centromere and defects in the mitotic spindle (Capitao et al., 2021). Nevertheless, despite the reduced level of cenH3, a very low frequency (0.2%) of haploids was found when this mutant was crossed with wild-type plants. Thus, the smaller centromere size did not efficiently trigger haploidization in *Arabidopsis* (Capitao et al., 2021). Considering the effect of heat stress on the *knl2* mutant, we measured HIR with *cenh3-4* mutants exposed to heat stress. We recapitulated the experiment using the same short-term stress conditions as for the *knl2* plants. The *cenh3-4* plants were subjected to 30°C for 2–3 days (Figure 1E), then pollinated with *gl1-1* pollen and cultivated for an additional 1–2 days at 30°C and 3 days at 25°C before transfer to STs. FC analysis of 56 seed pools (6 seeds per pool) with a total of 336 seeds revealed haploid and aneuploid induction rates of 3.3% and 2.7%, respectively (Table 1). A haploid induction frequency of 4.1% was determined on the basis of the trichomeless phenotype of *gl1-1* (Table 2). These results indicated that the *cenh3-4* mutation induces haploid formation under temperature stress, and sustaining this temperature stress for several days after pollination further improves HIR.

Finally, cenH3 RNAi transformants that revealed a substantial reduction of cenH3 at the centromeres (Lermontova et al., 2011) were tested as haploid inducers in combination with heat stress. Reciprocal crosses of RNAi with *gl1-1* plants were performed under short-term 30°C stress conditions (Figure 1E). In contrast to the results obtained with *cenh3-4*, FC analysis of these seeds did not reveal either haploids or aneuploids (Table 1).

## Discussion

In most eukaryotes, kinetochore assembly is primed by cenH3, and multiple kinetochore protein complexes are required for accurate chromosome segregation. We showed that disruption of the cenH3 loading machinery via the inactivation of the centromere licensing factor KNL2 of *Arabidopsis* resulted in the generation of haploids (HIR, 1%) upon outcrossing with the wild-type*.* To enhance the efficiency of haploid induction, we subjected the *knl2* mutant plants to various stress conditions such as increased temperature and light intensity because we found that deregulated expression of KNL2 leads to differential expression of many stress-responsive genes (Boudichevskaia et al., 2019). However, cultivation of *knl2* plants under long-term stress conditions (25°C/20°C day/night, 100 μmol m^−2^ s^−1^ light intensity or 21°C/18°C day/night and 400 μmol m^−2^ s^−1^ light intensity) did not increase HIR in the reciprocal crosses of *knl2* with the wild-type. Assuming that the applied cultivation regimens did not cause severe stress or that the plants had adapted to the long-term treatment, short-term treatment with high temperature (30°C/25°C day/night) was applied for 2–3 days prior to the crossing experiments and 1–2 days for the postfertilization process. Indeed, exposure of the *knl2* mutant to high temperatures for a short period enabled us to increase the HIR to up to 10%. This indicates that the haploid induction in centromere-impaired mutants is conditioned by temperature stress during both ovule development and early embryogenesis. Moreover, the same heat-stress treatment applied to the *cenh3-4* mutant (Capitao et al., 2021) resulted in an increase in HIR from 0.2% under STs to 4.1%.

**Figure 5:**
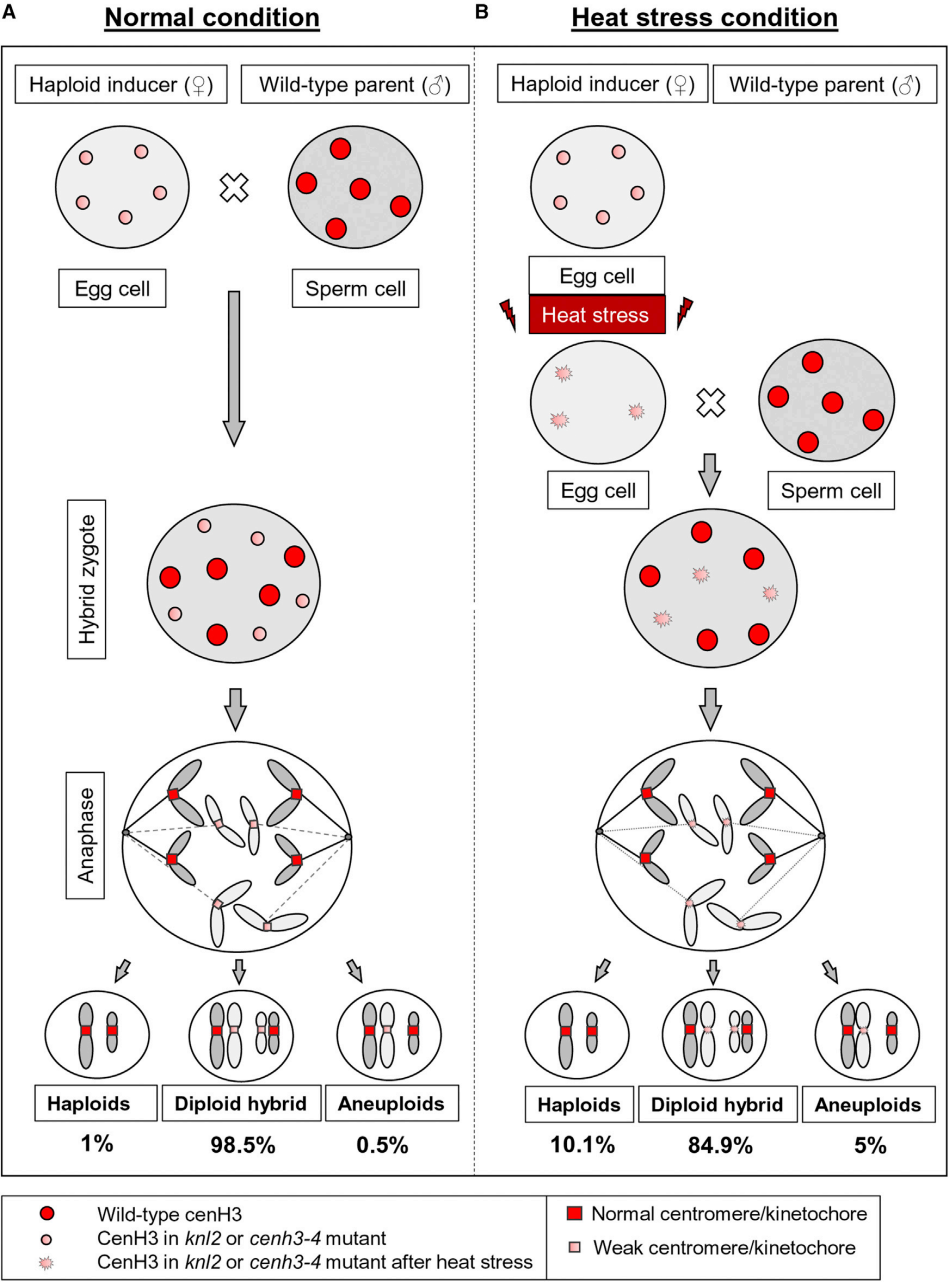
A model of haploid induction by uniparental chromosome elimination in crosses of the *knl2* and *cenh3-4* mutants with the wild-type under standard and heat-stress conditions. The model explains the elimination of uniparental chromosomes in haploid inducers (*knl2* or *cenh3-4* mutants) crossed with the wild-type under standard growth conditions **(A)** and heat-stress conditions **(B)**. **(A)** Under standard growth conditions, the combination of small centromeres of the haploid inducer (*knl2* or *cenh3-4* mutant) with “normal” wild-type centromeres in the hybrid zygote leads to centromere competition, followed by complete or partial elimination of the genome and formation of haploid (1%) and aneuploid (0.5%) progeny of the wild-type. **(B)** Short-term exposure of the haploid inducer (*knl2* or *cenh3-4*) to heat stress can further reduce cenH3 level, cause significant mitotic and meiotic abnormalities (Figure 4), and thus result in an HIR up to 10% when the inducer is crossed with wild-type plants.

Bioinformatics analysis revealed reduced expression of genes encoding kinetochore proteins, such as cenH3, KNL2, and CENP-C, under stress conditions (Supplemental Figure 1). These effects are not critical in the wild-type; however, in *knl2* and *cenh3-4*, they could amplify the effect of mutations. This suggestion is supported by our data showing that the frequency of mitotic and meiotic defects in *knl2* increases after short-term heat-stress, whereas wild-type plants cultivated under the same conditions did not show any mitotic or meiotic abnormalities. Our RNA-seq data analysis revealed that a large number of transposable elements were activated in seedlings and flower buds of the *knl2* mutant under standard growth conditions (Boudichevskaia et al., 2019). Thus, it is possible that exposure of *knl2* to heat stress may result in an increased number of active transposons and compromise chromatin organization (Probst and Mittelsten Scheid, 2015). In summary, we speculate that short-term temperature stress may cause the *knl2* and *cenh3-4* mutants to have smaller centromeres than under normal developmental conditions, which may enhance HIR (Figure 5). However, we would like to emphasize that this is merely a hypothesis; a clear mechanism remains to be elucidated because heat stress has complex effects on physiological, transcriptional, post-transcriptional, and epigenetic (DNA methylation, histone modification, and chromatin remodeling) processes (Zhao et al., 2020).

Originally, it was thought that haploids could be obtained only by crossing *cenh3* mutant lines complemented by a modified version of cenH3 with the wild-type (Ravi et al., 2014; Thondehaalmath et al., 2021). To understand the mechanism of genome elimination in such crosses, Marimuthu et al. (2021) analyzed the distribution of the altered cenH3 (GFP-tail swap) variant in gametes and at different developmental stages of hybrid zygotes. They observed that altered cenH3 is selectively removed from mature *Arabidopsis* eggs and early hybrid zygotes, whereas at the later zygotic stages, cenH3 and GFP-tail swap are preferentially loaded into the centromere of the wild-type parent. By contrast, cenH3-depleted mutant chromosomes are not able to reconstitute new cenH3 chromatin and undergo elimination. Wang et al. (2021) and Lv et al. (2020) have demonstrated that heterozygous cenH3 mutants of maize and wheat, respectively, can also function as efficient haploid inducers in crosses with the wild-type in both directions, despite the lack of competition between the two structurally different cenH3 variants. In these cases, it was assumed that weak centromeres are formed because of cenH3 dilution that occurs as a result of postmeiotic divisions in gametogenesis. Because female haploid spores undergo three mitotic divisions and males have only two, the level of cenH3 in female gametes is expected to be lower (Wang et al., 2021). When a heterozygous *cenh3-1* mutant of *Arabidopsis* was used as an haploid inducer in crosses with the wild-type, haploids were generated at a frequency of ∼1%, indicating that haploids can also be produced in *Arabidopsis* without alteration of cenH3 (Marimuthu et al., 2021).

Using *knl2* and *cenh3-4* mutants of *Arabidopsis* with reduced levels of cenH3, we further demonstrated that the use of structurally different variants of cenH3 is not the only way to generate haploids. Thus, as reported for maize and wheat, the centromere size model of Wang and Dawe (2018) can be applied to the haploid induction approach based on *knl2* and *cenh3-4* mutants.

It is important to understand why haploids cannot be obtained when heat-treated mutants are used as pollen donors. There are relatively few examples in which the effects of temperature stress on female reproductivity have been investigated, but much more is known about the effects of temperature stress on male reproductivity (Hedhly et al., 2009). Using tomato male-sterile and male-fertile lines, Peet et al. (1998) demonstrated that the application of stress to pollen donor plants before and during pollen release decreased seed number and fruit set more severely than heat stress applied to the developing ovule. Thus, heat-stress treatment of *knl2* and *cenh3-4* mutants as pollen donors can lead to a decrease in pollen viability and the inability to fertilize the egg. At the same time, fertilization of the ovule with viable pollen will not lead to the process of genome elimination. By contrast, heat-stress treatment of mutants as maternal crossing partners leads only to a weakening of the centromeres without affecting viability of the ovules.

The most notable male meiosis phenotypes in the *knl2* mutant were the presence of lagging chromosomes and fragmentation. Lagging chromosomes could appear as a consequence of weaker or defective centromere activity in *knl2* plants. Fragmentation appeared during mitosis and meiosis. Interestingly, two plants grown at 30°C showed fragmentation in anthers during the first meiotic division, and the other two plants showed fragmentation during the second meiotic division. This suggests that chromosome fragments could originate from different mechanisms. One possibility is that the interlocks observed during pachytene are not properly repaired. Another possibility is defective DNA repair in pathways involving both inter-sister and inter-homolog events. Anaphase bridges were also observed because of the effect of heat stress on the *knl2* mutant. Our comparative analysis of seedling and flower bud transcriptomes from the *knl2* mutant and wild-type showed that genes encoding proteins involved in DNA repair were overrepresented among downregulated *knl2* genes (Boudichevskaia et al., 2019). For instance, the downregulated genes encoded KU70 (AT1G16970), KU80 (AT1G48050), and LIGASE 4 (AT5G57160), key players that participate in canonical non-homologous end joining; RAD51 (AT5G20850), which is essential for meiotic repair of DSBs caused by AtSPO11-1 (Li et al., 2004); DMC1 (AT3G22880), known to promote interhomolog recombination; and SMC6A (At5G07660) and SMC6B (At5G61460), two components of the SMC5/6 complex engaged in DNA repair, meiotic synapsis, genome organization, and stability. Previously, high temperatures were shown to disturb genome integrity by causing strand breakages and impeding DNA repair (Kantidze et al., 2016), and crosstalk also exists between heat and genotoxic stress (Han et al., 2020). We speculate that because of the reduced expression of genes encoding components of the DNA-repair mechanism, the *knl2* mutant cannot cope with heat-induced DNA damage as efficiently as the wild-type, leading to increased mitotic and meiotic defects in *knl2* after exposure to high temperature.

In principle, we would expect that cenH3 RNAi transformants with strongly reduced cenH3 levels would also work as haploid inducers (Lermontova et al., 2011). However, subjecting our cenH3 RNAi transformants to heat stress did not result in haploid formation when they were crossed with the untreated wild-type. Previously, it was shown that cenH3 levels in the cenH3 RNAi transformants were more strongly reduced in leaves than in root tips enriched in meristematic cells (Lermontova et al., 2011). On the basis of previously published data, we hypothesized that this may be because of decreased activity of the cauliflower mosaic virus (CaMV) 35S promoter used (Holtorf et al., 1995; Wang et al., 2015) and suppression of post-translational gene silencing in meristems and reproductive tissues, which may decrease the function of the RNAi machinery (Mitsuhara et al., 2002). Using maize cenH3 RNAi lines complemented with the *AcGREEN-tail swap-CENH3*, Kelliher et al. (2016) demonstrated that these lines generated 0.24% maternal and 0.07% paternal haploids when crossed with the wild-type. Thus, haploid induction through the reduction of cenH3 levels by expressing cenH3 RNAi constructs appeared to be inefficient in *Arabidopsis* and maize.

The introduction of point mutations in cenH3 or in the conserved CENPC-k motif of KNL2 is sufficient to generate haploid-inducer lines. Thus, the use of *knl2* and *cenh3* mutants obtained via chemical ethyl methanesulfonate mutagenesis could circumvent the use of transgenic plants for haploid production. Alternatively, mutants can be produced by targeted mutagenesis using the CRISPR-Cas9 approach. In either case, complementation with altered cenH3 variants is not required, making the production of haploid inducers much easier. Moreover, under standard growth conditions, the growth rate of the *cenh3-4* mutant is similar to that of the wild-type, and the growth rate of *knl2* is slightly reduced. Thus, we believe that obtaining vigorous haploid inducers and their short-term exposure to heat stress before crossing with the wild-type have great potential in plant breeding.

## Methods

### Plasmid construction, plant transformation, and plant growth conditions

To analyze whether complementation of the *knl2* mutant with the genomic KNL2:KNL2-EYFP fusion construct would abolish the ability of *knl2* to induce haploids, we amplified the genomic *KNL2* fragment (464 up to +2513 relative to the transcriptional *KNL2* start site) by PCR from Col-0 genomic DNA using KNL2-attB1gensh and KNL2-attB2 primers (Supplemental Table 2) and cloned it into the pDONR221 vector. These constructs were used to generate the KNL2:KNL2-EYFP fusion construct using the pGWB640 vector (https://novoprolabs.com/vector/Vgy4dmna). Substitution of the conserved amino acid Trp by Arg within the CENPC-k motif of KNL2 was performed by PCR using the Phusion site-directed mutagenesis kit (Thermo Fisher Scientific). A KNL2:KNL2-EYFP/pDONR221 construct was PCR mutagenized using the following primer pairs: KNL2gen_W_R_f and KNL2gen_W_R_r for the substitution of Trp by Arg (Supplemental Table 2). *A. thaliana* plants were transformed by the floral dip method (Clough and Bent, 1998). T1 transformants were selected on Murashige and Skoog medium containing 20 mg l^−1^ phosphinothricin. The plants were propagated under short- or long-day conditions in a cultivation room at 8 h light/20°C:16 h dark/18°C and 16 h light/20°C:8 h dark/18°C, respectively.

### Immunostaining and microscopy analysis of fluorescent signals

Immunostaining of nuclei/chromosomes was performed as described previously (Sandmann et al., 2016). CenH3 was detected in flower bud nuclei with rabbit anti-CENH3 primary antibody (1:1000 dilution) and the secondary antibody rhodamine-conjugated anti-rabbit IgG (111-295-144; Jackson). Wide-field fluorescence microscopy was used to evaluate and image the nuclei preparations with an Olympus BX61 microscope (Olympus, Tokyo, Japan) and an ORCA-ER CCD camera (Hamamatsu, Japan). For live-cell imaging, *Arabidopsis* seeds of lines harboring mutagenized KNL2:KNL2-EYFP/pGWB640 variants or non-mutagenized controls were germinated in agar medium in coverslip chambers (Nalge Nunc). Roots growing parallel to the coverslip bottom were analyzed with an LSM 510META confocal microscope (Carl Zeiss) using a 63× oil immersion objective (NA 1.4). EYFP was excited with a 488-nm laser line, and fluorescence was recorded with a 505- to 550-nm band-pass filter. Images were analyzed with LSM software release 3.2.

### Whole-mount preparation

Siliques of different developmental stages were fixed in ethanol:acetic acid (9:1) overnight at 4°C and dehydrated in 70% and 90% ethanol for 1 h each. The preparation was then cleared in chloral hydrate (chloral hydrate:water:glycerol = 8:2:1) overnight at 4°C. Seeds in siliques were counted under a binocular microscope (Carl Zeiss, Germany).

### Cytogenetic techniques

*A. thaliana* inflorescences were fixed in freshly prepared ice-cold ethanol:acetic acid (3:1) and stored at 4°C for chromosome preparations by the spreading technique (Armstrong et al., 2009). After cell-wall digestion, individual buds were dissected on slides, treated with 60% acetic acid, and spread for 30 s on a hot plate at 45°C, stirring the meiocyte suspension. Post-fixation was performed by applying ice-cold ethanol:acetic acid (3:1). Air-dried slides were counterstained with DAPI and mounted in VECTASHIELD medium. Images were acquired with a Nikon Eclipse Ni microscope equipped with a Nikon DS-Qi2 camera and NIS Elements v.4.60 software.

### SNP analysis

Next-generation sequencing of genomic DNA was performed by Eurofins Genomics Europe Shared Services GmbH (Konstanz, Germany) using the Illumina NovaSeq 6000 sequencing platform with approximately 5 × 10^6^ reads per sample. The data are openly available in European Nucleotide Archive (ENA) at https://www.ebi.ac.uk/ena/browser/view/PRJEB58839. After adapter trimming with cutadapt v.1.15 (Martin, 2011), the reads were aligned to the TAIR10 assembly with minimap2 v.2.17 (Li, 2018). Alignment records were converted to BAM format with SAMtools (Li et al., 2009) and sorted with NovoSort (http://www.novocraft.com/products/novosort/). SNP calling was performed with BCFtools v.1.9 (Li, 2011) (command ‘mpileup’ and ‘call’) using the parameters ‘-a DP,DV’ to record allelic depths. Only reads with a mapping quality ≥Q20 were considered for variant calling. Allelic depths for each sample at bi-allelic SNP sites with a quality score ≥Q40 were written to tabular format and read into R (R Core Team, 2017) for further processing. Homozygous genotype calls for the reference (alternative) allele were made if ≤10% (≥90%) of reads supported the variant allele. Heterozygous calls were made if 40%–60% of reads supported the variant allele. If allelic ratios were outside these ranges or the total read depth was <5, genotype calls were set to missing. SNPs at which the parents carried opposite homozygous alleles were selected to plot graphical genotypes of the progeny along the genome.

### Gene expression analysis

Transcriptome data were retrieved from the *Arabidopsis* RNA-seq Database (available at: http://ipf.sustech.edu.cn/pub/athrdb/) (Zhang et al., 2020). The gene expression profiles of *cenH3*, *CENP-C*, *KNL2*, and *NASP* were extracted for different stress treatments, including temperature and light stress. Downregulated treatments among these genes were used for comparative co-expression analysis.

### FC ploidy measurements of seeds

To measure seed ploidy, we chopped together six seeds per pool in 500 μl nuclei isolation buffer (Galbraith et al., 1983) supplemented with propidium iodide (PI; 50 μg/ml) and DNase-free RNase (50 μg/ml) in a Petri dish using a sharp razor blade. The resulting nuclei suspensions were filtered through a 50-μm mesh (CellTrics; Sysmex Partec) and measured on a CyFlow Space flow cytometer (Sysmex Partec), a FACSAria cell sorter (BD Biosciences), or a BD Influx cell sorter (BD Biosciences). The presence of haploid/aneuploid seeds within the pools was determined by evaluating the PI fluorescence intensity on a linear scale. Because the precise number of haploids/aneuploids per pool cannot be determined unequivocally, we considered only one seed per pool as deviating from the diploid status if an additional peak was found.

## Funding

This research was supported by the 10.13039/501100002347German Federal Ministry of Education and Research (Plant 2030; Project 031B0192NN, HaploTools), the 10.13039/501100001659Deutsche Forschungsgemeinschaft (LE2299/3-1 and LE2299/5-1), and the 10.13039/501100008530European Regional Development Fund-Project “REMAP” (CZ.02.1.01/0.0/0.0/15_003/0000479) to K.R.

## Author contributions

U.A. and M.K. contributed equally to this work. I.L., K.R., A.H., and M.K. conceived the study and designed the experiments. I.L., U.A., M.K., L.C.K., J.F., M.C., D.D., S.Z., J.P., M.M., and S.H. performed the experiments. U.A., M.K., I.L., and K.R. wrote the manuscript. All authors read and approved the final manuscript.

## Supplemental information

Document S1. Supplemental Figures 1–6 and Supplemental Tables 1 and 2

Document S2. Article plus supplemental information
