# Supplementary material for: High temperature increases centromere-mediated genome elimination frequency and enhances haploid induction in Arabidopsis
Source: Plant Commun. 2022 Dec 20;4(3):100507. doi: 10.1016/j.xplc.2022.100507 (PMC10203384; doi:10.1016/j.xplc.2022.100507)
Supplement: Document S1. Supplemental Figures 1–6 and Supplemental Tables 1 and 2 [file mmc1.pdf]

Plant Communications, Volume *4*

Supplemental information

High temperature increases centromere-mediated genome elimination frequency and enhances haploid induction in *Arabidopsis*

Ulkar Ahmadli, Manikandan Kalidass, Lucie Crhak Khaitova, Joerg Fuchs, Maria Cuacos, Dmitri Demidov, Sheng Zuo, Jana Pecinkova, Martin Mascher, Mathieu Ingouff, Stefan Heckmann, Andreas Houben, Karel Riha, and Inna Lermontova

Plant Communications

Supplemental information

High temperature increases centromere-mediated genome elimination frequency and enhances haploid induction in *Arabidopsis*

Ulkar Ahmadli, Manikandan Kalidass, Lucie Crhak Khaitova, Joerg Fuchs, Maria Cuacos, Dmitri Demidov, Sheng Zuo, Jana Pecinkova, Martin Mascher, Mathieu Ingouff, Stefan Heckmann, Andreas Houben, Karel Riha and Inna Lermontova

Supplemental information

High temperature increases centromere-mediated genome elimination frequency and enhances haploid induction in *Arabidopsis*

Ulkar Ahmadli[1#], Manikandan Kalidass[1#], Lucie Crhak Khaitova[2], Joerg Fuchs[1], Maria Cuacos[1], Dmitri Demidov[1], Sheng Zuo[2], Jana Pecinkova[2], Martin Mascher[1], Mathieu Ingouff[3], Stefan Heckmann[1], Andreas Houben[1], Karel Riha[2] and Inna Lermontova[1]

[1]Leibniz Institute of Plant Genetics and Crop Plant Research (IPK) Gatersleben, Corrensstrasse 3, D-06466 Seeland, Germany

[2]Central European Institute of Technology (CEITEC) and National Centre for Biomolecular Research, Faculty of Science, Masaryk University, Kamenice 5, 625 00 Brno, Czech Republic

[3]CIRAD, DIADE, IRD, University of Montpellier, 34393 Montpellier, France

*Correspondence: lermonto@ipk-gatersleben.de

# - contributed equally to this work

The supplemental file includes 6 Supplemental Figures and 2 Supplemental Tables

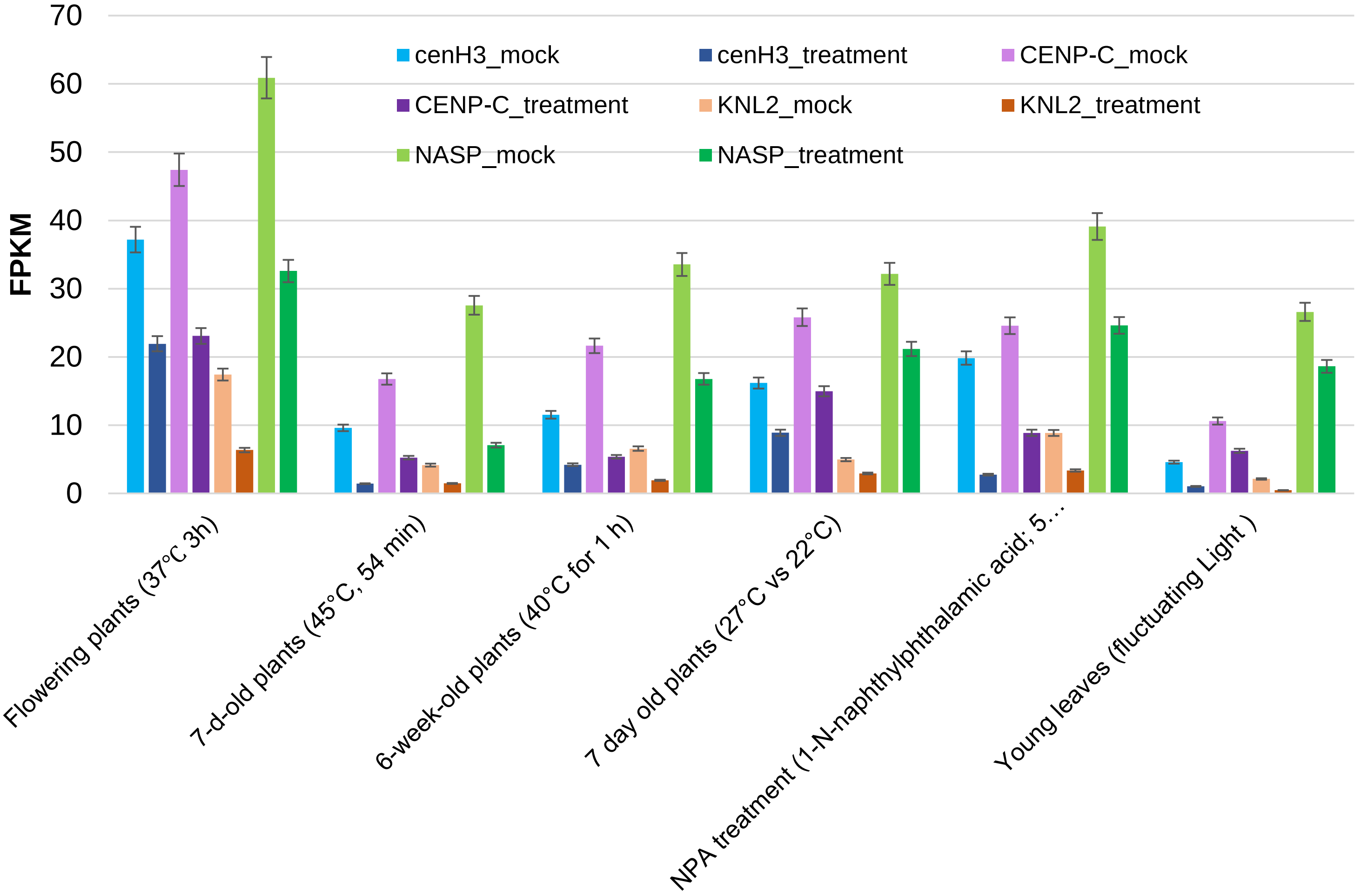

Supplemental Figure 1. Expression profiles of cenH3 and other genes encoding kinetochore proteins under different stress conditions in *A. thaliana.*

Comparative transcriptome analysis showed gene expression difference (shown in different colors) under different stress treatment (x-axis) in *Arabidopsis*. Fragments Per Kilobase of transcript per Million mapped reads is the abbreviation for FPKM (y-axis) values used for expression analysis. In RNA-Seq, a transcript's relative expression is proportional to the number of cDNA fragments it generates. Bar graph showed that heat treatment has leaded downregulated expression of cenH3, CENP-C, KNL2 and NASP. The transcriptional dataset was download under accession numbers: PRJNA363056; PRJDB7363; PRJNA317804; PRJNA497220; PRJNA489360; PRJEB31094. Error bars corresponds to SEM.

knl2 WT Col-0 *gl1-1 Ler*

A

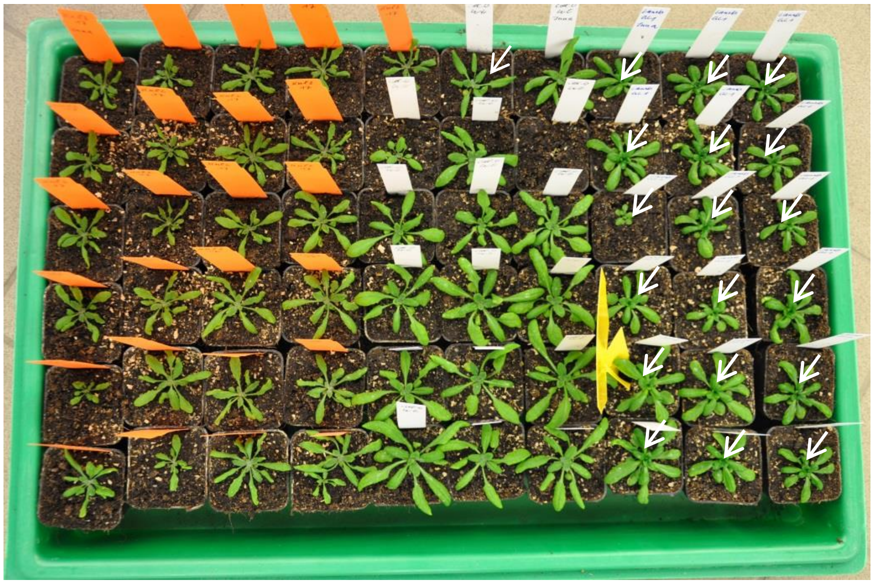

Standard conditions (21/18°C),

100 µmol m^{-2} sec^{-1} **(ST)**

knl2 WT Col-0 *gl1-1 Ler*

B

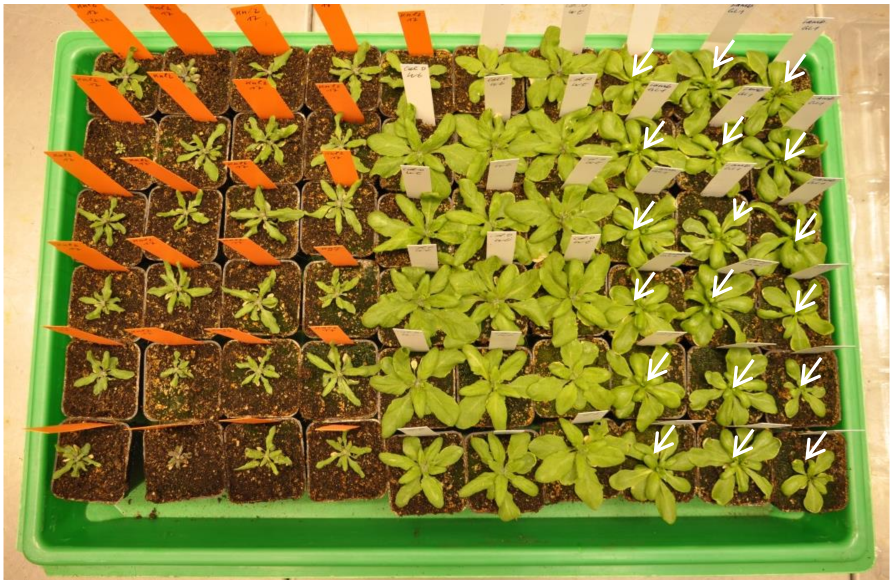

High temperature (25/21°C),

100 µmol m^{-2} sec^{-1} **(HT)**

knl2 WT Col-0 *gl1-1 Ler*

C

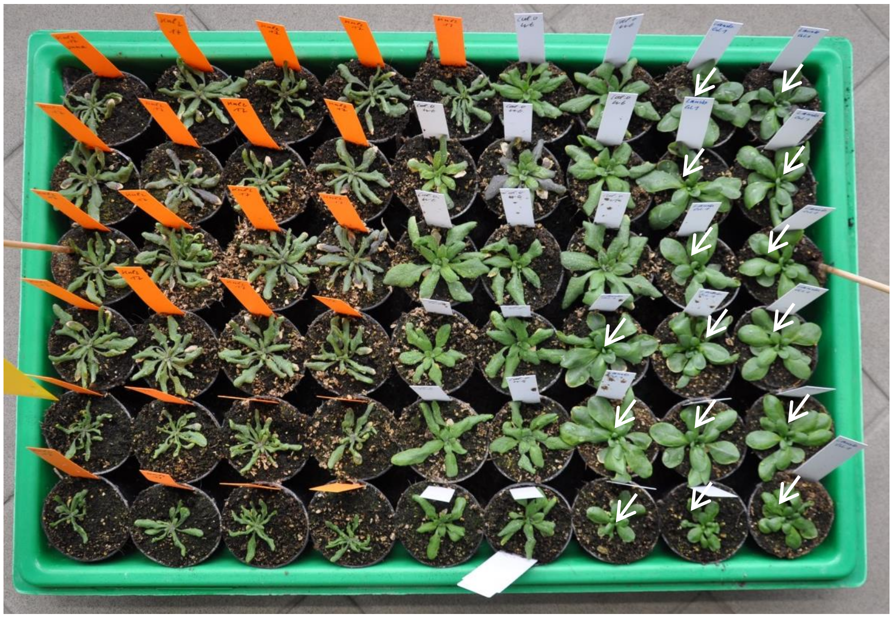

High light (21/18°C),

400 µmol m^{-2} sec^{-1} **(HL)**

Supplemental Figure 2. Phenotype of 5-week-old *knl2*, wild-type (WT Col-0) and *gl1-1* of *L. erecta* cultivated under different growth conditions.

All plants were cultivated under standard growth conditions (21/18°C day-night and light intensity 100 µmol $m^{-2}sec^{-1}$) for three weeks, then some plants were left under standard conditions **(A)** or transferred to higher temperature (25/21°C day night) **(B)** or higher light intensity (400 µmol $m^{-2}sec^{-1}$) **(C)** conditions. Orange labels were used to mark *knl2*, while white labels marked *A. thaliana* wild-type Col-0 and the trichome-less *gl1-1* mutant of *L. erecta* (additionally shown by white arrows).

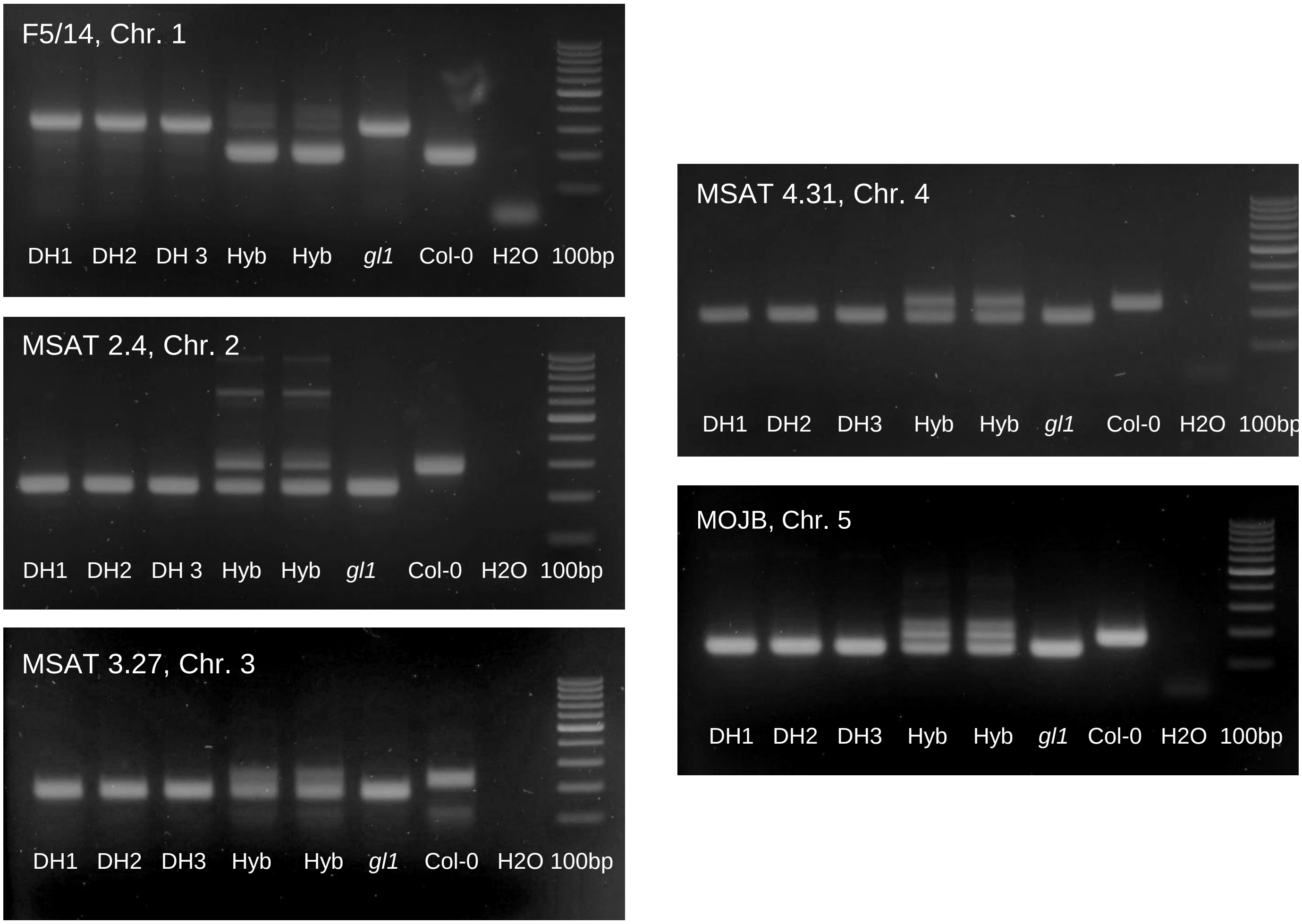

Supplemental Figure 3. Band patterns of chromosome markers amplified on template DNA isolated from double haploids (DH), F1 heterozygous progeny (Hyb) and the parental *gl1-1* (Ler) and Col-0 plants.

PCR-amplified DNA fragments were electrophoresed on 2.5 % agarose gels, together with size marker Gene Ruler 100bp (Thermo Scientific) on the right side. The chromosome number is shown inside of each panel.

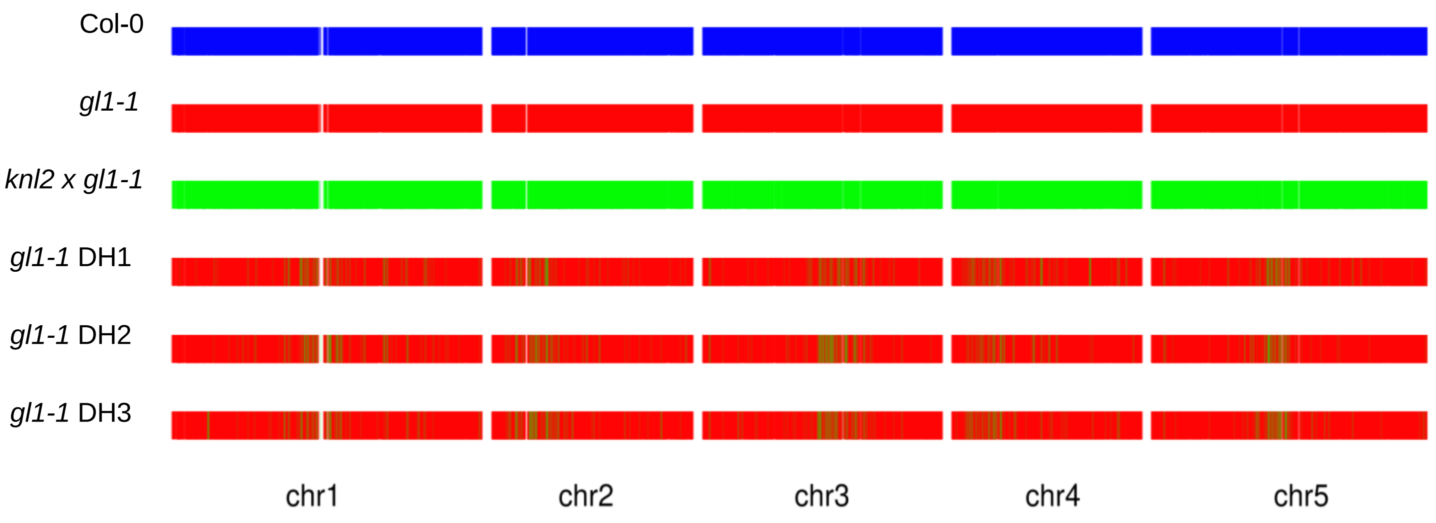

Supplemental Figure 4. Confirmation of haploid progeny obtained by crossing of *knl2* with the trichome-less *gl1-1* mutant.

Single Nucleotide Polymorphism (SNP) analysis of three *gl1-1* double haploids, *knl2 x gl1-1* hybrid, *gl1-1,* and Col-0 plants. The results displayed that the hybrid plants were completely heterozygous whereas *gl1-1* double haploid plants do not contain any residues of the maternal *knl2* mutant genome.

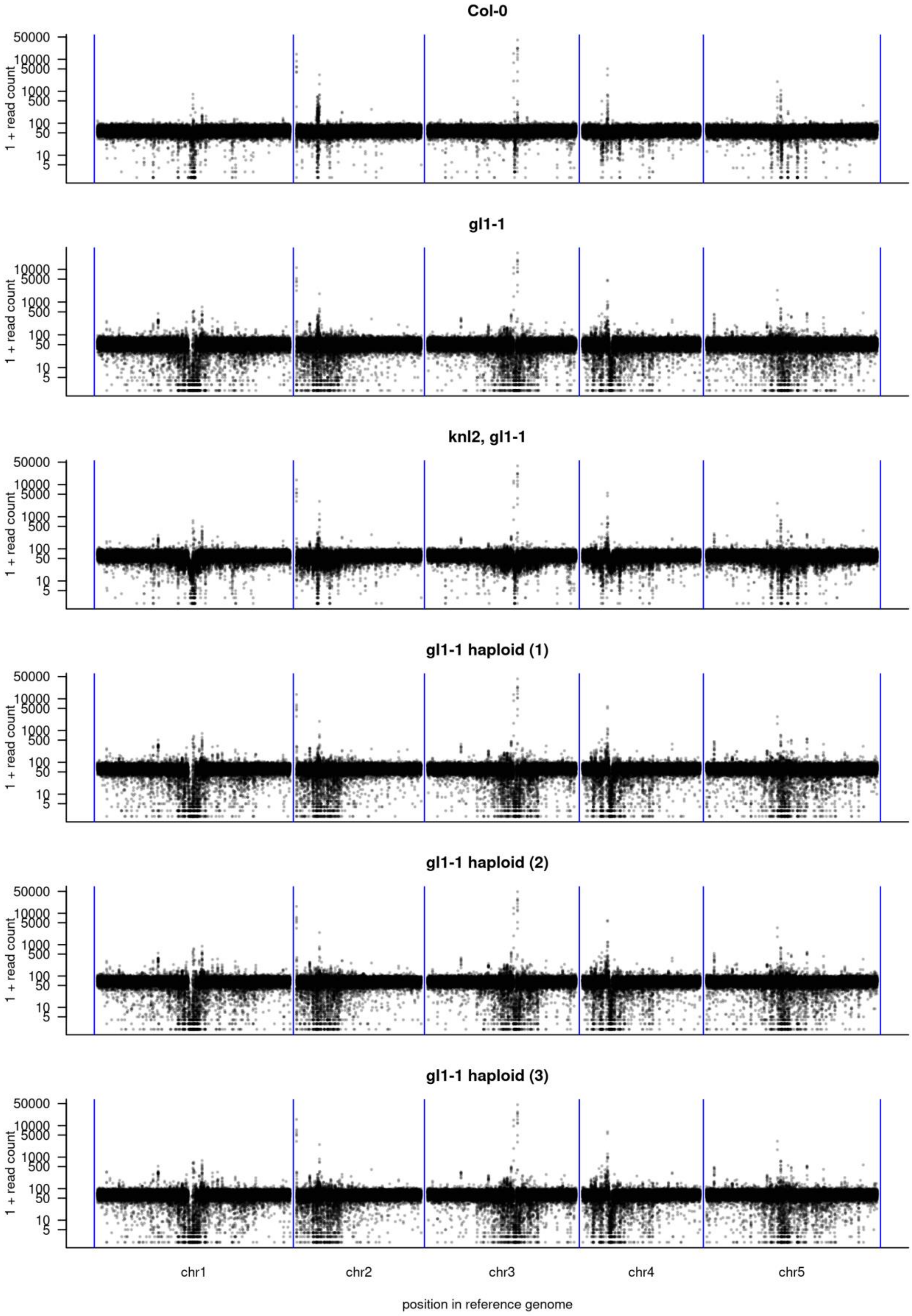

Supplemental Figure 5. Read depth in *gl1-1,* Col-0, a *knl2* x *gl1-1* hybrid and three *gl1-1* haploid plants.

The number of uniquely mapped reads (MAPQ20) in 10 kb windows along the TAIR10 assembly is shown. Due to difficulties in mapping short-reads to repetitive regions coverage around centromeres is variable. No large deletions or duplications private to the *gl1-1* haploids were observed.

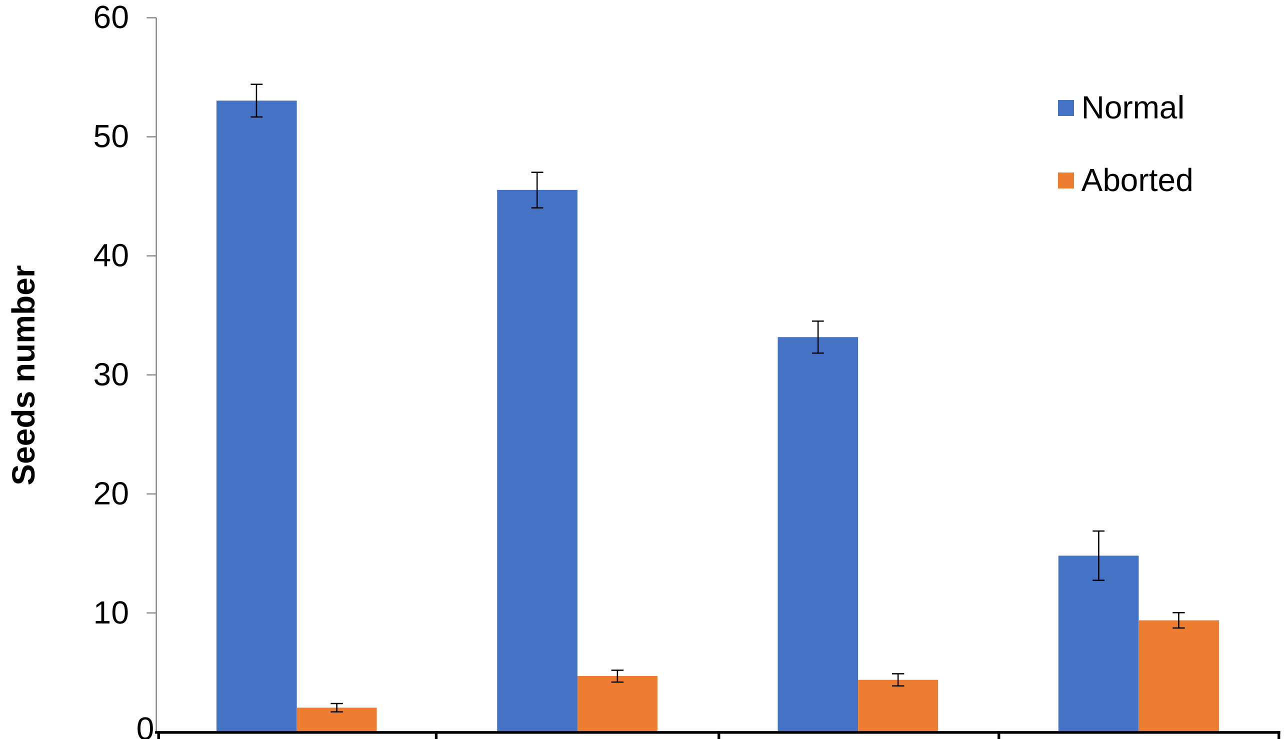

	Col-0 ST	*knl-2* ST	Col-0 30°C	*knl-2* 30°C
Normal	53.04	45.53	33.17	14.81
Aborted	2.04	4.70	4.40	9.39

Supplemental Figure 6. Exposure of *Arabidopsis knl2* mutant and wild-type to high temperature resulted in a decreased seed setting and an increased number of aborted seeds.

Seed setting analysis was performed on selfed plants either continuously grown under standard growth conditions or exposed for 4 days to 30/25°C (day/night) as it is indicated in Figure 1E. Normal and aborted seeds has been calculated for Col-0 and *knl2* mutant plants under standard and heat stress conditions. Graph bar shows that aborted seeds were higher in heat stressed *knl2* mutant compared to Col-0. Error bar corresponds to SEM.
